# Do parents who smoke underutilize health care services for their children? A cross sectional study within the longitudinal PIAMA study

**DOI:** 10.1186/1472-6963-7-83

**Published:** 2007-06-12

**Authors:** Monique AM Jacobs-van der Bruggen, Alet H Wijga, Bert Brunekreef, Johan C de Jongste, Caroline A Baan, Marjan Kerkhof, Henriette A Smit

**Affiliations:** 1 National Institute of Public Health and the Environment (RIVM), Bilthoven, The Netherlands; 2 Institute for Risk Assessment Sciences, University of Utrecht, Utrecht, The Netherlands; 3 Department of Pediatrics, Erasmus University Medical Center, Sophia Children's Hospital, Rotterdam, The Netherlands; 4 Department of Epidemiology, University Medical Center Groningen, University of Groningen, The Netherlands

## Abstract

**Background:**

A higher prevalence of respiratory symptoms and an associated increase in health care utilization among children with parents who smoke is to be expected. From previous studies however, it appears that parents who smoke may underutilize health services for their children, especially with respect to respiratory care. This study explores the validity and generalizability of the previous assumption.

**Methods:**

Data were obtained from a Dutch birth-cohort study; the Prevention and Incidence of Asthma and Mite Allergy (PIAMA) project. Information regarding parental smoking, the child's respiratory symptoms and health care use and potential confounders were obtained by postal questionnaires. Multivariate logistic models were used to relate parental smoking to the child's respiratory symptoms and health care use.

**Results:**

The study comprised 3,564, 4-year old children. In the crude analysis, respiratory symptoms were more frequent among children with a parent who smoked, while health care utilization for respiratory symptoms was not significantly different between children with or without a parent who smoked. In the multivariate analyses, maternal smoking had a larger impact on the child's respiratory symptoms and health care use as compared to paternal smoking. Maternal smoking was positively associated with mild respiratory symptoms of the child, adjusted odds ratio [AOR] 1.50 (1.19–1.91), but not with severe respiratory symptoms AOR 1.03 (0.75–1.40). Among children with mild respiratory symptoms, children with a mother who smoked were less likely to be taken to the general practitioner (GP) for respiratory symptoms, than children with mothers who did not smoke, AOR 0.58 (0.33–1.01). This finding was less pronounced among children with severe respiratory symptoms AOR 0.86 (0.49–1.52). Neither GP visits for non-respiratory symptoms nor specialized care for respiratory disease were significantly associated with parental smoking.

**Conclusion:**

Mothers who smoke appear to underutilize health care for their children with mild respiratory symptoms. Health care workers should be informed about this phenomenon. Inquiring after the respiratory health of the children during regular visits to healthy baby clinics may help to track potential underutilization of care.

## Background

It is fairly well established that exposure to environmental tobacco smoke (ETS) is associated with an increased prevalence of respiratory symptoms in young children [1-7]. It is unclear whether this increased frequency of symptoms translates into higher use of different health care services. In some studies among children, ETS exposure was positively associated with physician visits [8], need for asthma medication [9,10], hospitalization [10-12] and emergency care [13,14]. However, some studies did not find significant associations between ETS and physician visits [15], hospital admission rates [10,14-16] and in- and outpatient care for respiratory illness [17]. Finally, ETS exposure appeared negatively associated with preventive care [15,17], hospital visits [8], and physician visits for asthma [18].

Exposure to ETS in young children is mainly determined by parental smoking. It is hypothesized that parents who smoke may underutilize health services for their children, or use services differently from parents who do not smoke [15]. They might either visit a physician for different reasons, or postpone visits until the symptoms of their children are more severe. In order to explore whether parents who smoke underutilize health care for their children, it is important whether or not the associations -between parental smoking and health care use of the child- are adjusted for the increased prevalence (or severity) of respiratory symptoms in children from parents who smoke. In a study among asthmatic children, maternal smoking as compared to paternal smoking only, was independently related to fewer physician visits for asthma for the children [18]. This association was independent of adjustment for asthma severity. For visits related to non-respiratory symptoms, the association seemed to be opposite. Crombie et al. [18] suggest a possible lack of awareness of asthma symptoms among heavy smokers or a reluctance of parents who smoke to visit the physician for asthma-related symptoms.

Our study comprises parents who smoke and parents who do not smoke and children with and without doctor diagnosed asthma. In this cohort we explore the associations between parental smoking and the child's respiratory symptoms and health care use. We expect that maternal smoking has a larger impact on the child's respiratory symptoms and health care use than paternal smoking, because mothers generally spent more time with the children and are probably more likely to take the child to the doctor than the fathers. We specify our results according to the reason for the health care visit (respiratory- or non-respiratory) and we adjust the associations between parental smoking and health care use for the, probably increased, prevalence of respiratory symptom in children from parents who smoke.

## Methods

Data were collected in the Prevention and Incidence of Asthma and Mite Allergy (PIAMA) project. PIAMA is a longitudinal Dutch birth cohort study of 4,146 children born between July 1996 and October 1997. In the PIAMA project, data were collected prenatal, at three months, at age one, and yearly thereafter by means of postal surveys which are filled out by one of the parents. This paper focuses on health care utilization of four year old children in relation to smoking behavior of their parents in the previous year.

Parents filled in whether they visited the general practitioner in the past two months and if yes, how often and for what reason:

asthma, shortness of breath or wheezing

skin problems or eczema

allergy or hay fever

other reasons such as:

Self-reported information was also obtained for visits to several kinds of medical specialists in the past two months and (reasons for) hospital admissions in the past year. Information on characteristics of the children and parents was obtained from the earlier surveys. Methods of the PIAMA study have been described in more detail elsewhere [19].

There were 4,146 children included in the PIAMA study. For 3,564 children (85.9%) information was available from the baseline- and the three- and four-years questionnaires. The study sample for this study comprises 3,564 children with a mean age of 49 months.

Outcome variables were defined as whether or not the parent visited the general practitioner (GP visit) for the child in the past two months for:

1. any reason,

2. respiratory symptoms (shortness of breath, wheezing, cough, common cold, head cold, asthma, pneumonia, bronchitis, the flu, fever, ear-, nose- or throat problems), or

3. only non-respiratory symptoms (all other reasons).

Specialized care for respiratory symptoms was defined as a visit to a (child) pneumonologist or ear-, nose- or throat specialist in the past to months or a hospital admission for asthma, bronchitis, pneumonia or ear -, nose- or throat problems in the past year.

Determinants were defined as:

1. at least one parent who smoked at home,

2. a mother who smoked at home,

3. a father who smoked at home,

We used smoking data of 1 year previous to the measures of respiratory symptom severity and health care use by the children.

Potential confounders were defined as birth weight (g.), gender, doctor diagnosed asthma ever, number of older siblings, whether the child was breastfed for at least 8 weeks or not, age of each parent, allergic constitution of each parent (yes/no), ethnicity of each parent (born in Netherlands and Dutch ethnicity, born in Western country and Dutch or Western ethnicity, not born in Western country or non-Western ethnicity), and educational level of each parent (seven point scale, in which a higher number corresponds to a higher level of education). Severity of the child's respiratory symptoms was defined as:

1. no respiratory symptoms (see 2 and 3),

2. mild respiratory symptoms: wheezing 1 or 2 attacks in the past year or shortness of breath 1 or 2 attacks in the past year or "regular congested chest or cough with phlegm with cold" in the past year, or airway infection (including nose, and throat problems) 3 to 5 times in the past year, no severe respiratory symptoms,

3. severe respiratory symptoms: wheezing at least 3 attacks in the past year or shortness of breath at least 3 attacks in the past year or "regular congested chest or cough with phlegm, without cold" or "congested chest or cough with phlegm on at least 4 days per week during at least 3 months in the past year" or airway infection at least 6 times in the past year, or asthma, bronchitis or doctor diagnosed pneumonia in the past year.

Differences in population characteristics and outcome variables between children with and without at least one parent who smoked were analyzed with Chi-squared tests for categorical variables and t-tests for continuous variables. The associations between parental smoking, and the child's respiratory symptoms and health care use were analyzed with logistic regression models. In the multivariate models for respiratory symptoms we adjusted for doctor diagnosed asthma ever (yes/no), gender of the child, birth weight (continuous), number of older siblings (continuous), child being breastfed for at least 8 weeks (yes or no), age of each parent (continuous), ethnicity of each parent (categorical), education of each parent (continuous), and allergic constitution of each parent (yes or no). Multivariate models for health care use were additionally adjusted for the severity of the child's respiratory symptoms (categorical as no, mild or severe).

All variables had less then 3% missing values. Missing values were set to zero; 'factor not present' (dichotomous variables), included as a separate category (ethnicity) or replaced with mean values (continuous variables). The reason for visiting the GP (respiratory or non-respiratory) was unknown for 3 children and these children were only included in the analysis regarding GP visits for any reason. For all analyses a level of significance of p < 0.05 was applied. All analyses were performed with SAS version 9.1 (SAS Institute Inc., Cary, NC, USA).

## Results

General characteristics of the children in relation to parental smoking are described in table 1. About 23% of the children (805 children) had at least one parent who smoked at home; 473 children had a mother who smoked at home and 613 children had a father who smoked at home (correlation between smoking by the mother and the father r = 0.44). Children with or without at least one parent who smoked differed with respect to birth weight of the child, maternal age, breastfeeding, allergic constitution and education of the parents and ethnicity of the father.

**Table 1:** Population characteristics in relation to parental smoking

	total n = 3,564	no parent smokes n = 2,759	at least one parent smokes n = 805	
mean birth weight of the child (gr.)	3515 (543)	3536 (534)	3444 (568)	*
mean age of the mother (sd)	30.5 (3.8)	30.6 (3.8)	30.2 (4.1)	*
mean age of the father (sd)	32.8 (4.5)	32.9 (4.5)	32.7 (4.7)	
male gender of the child (%)	51.7	51.8	51.2	
mean number of older siblings	0.70 (0.87)	0.69 (0.87)	0.74 (0.87)	
% breastfed for at least 8 weeks	60.9	65.1	46.6	*
% with allergic mother	28.9	29.9	25.5	*
% with allergic father	30.5	32.2	24.7	*
education mother (%)				
*low (level 1 or 2)*	12.1	9.2	22.0	*
*middle (level 3,4 or 5)*	52.0	50.4	57.4	*
*high (level 6 or 7)*	35.1	39.7	19.4	*
education father (%)				
*low (level 1 or 2)*	18.4	15.5	28.1	*
*middle (level 3,4 or 5)*	40.4	38.8	45.8	*
*high (level 6 or 7)*	39.4	44.0	23.6	*
ethnicity mother (%)				
*born in Netherlands and Dutch ethnicity*	92.4	92.6	91.8	
*born in Western country and Dutch or Western ethnicity*	1.9	2.0	1.6	
*not born in Western country or non-Western ethnicity*	3.2	3.0	4.1	
ethnicity father (%)				
*born in Netherlands and Dutch ethnicity*	91.1	91.8	88.5	*
*born in Western country and Dutch or Western ethnicity*	1.8	1.7	2.2	
*not born in Western country or non-Western ethnicity*	4.4	3.8	6.3	*

sd: standard deviation

* significant difference between no smoking parent or at least one smoking parent; p < 0.05

The results for respiratory symptoms and health care use in relation to parental smoking are summarized in table 2. The percentage of children with respiratory symptoms was significantly higher among children with at least one parent who smoked as compared to children with parents who did not smoke (47.1% versus 41.6%, p = 0.006). GP visits were made by 1,068 children; 512 children visited the GP for respiratory symptoms and 553 children visited for non-respiratory symptoms only (for 3 children, the reason for the visit was unknown). Specialized care for respiratory symptoms was used by 304 children; 290 children visited a medical specialist and 31 children were admitted to a hospital. There were no significant differences in health care use between children with or without a parent who smoked.

**Table 2:** Respiratory symptoms and health care use for children with and without a parent who smokes.

	no parent smokes n = 2,759	at least one parent smokes n = 805
**Respiratory symptoms**		
any respiratory symptoms	41.6	47.1 *
mild respiratory symptoms	24.5	30.6 *
severe respiratory symptoms	17.0	16.5
**Health care use**		
GP visits for any reason	29.7	30.9
GP visits for respiratory symptoms	14.3	14.8
GP visits for non-respiratory symptoms only	15.4	16.2
Specialized care for respiratory symptoms	8.4	8.9

In the multivariate analysis the severity of the child's respiratory symptoms was a strong and independent determinant of health care use for respiratory symptoms. Results of the multivariate analysis for parental smoking, respiratory symptoms and health care use are summarized in table 3. As expected, maternal smoking had a larger impact on the child's respiratory symptoms and health care use than paternal smoking. The frequency of mild respiratory symptoms was substantially increased if the mother smoked AOR 1.50 (1.19–1.91). With respect to health care use, the only significant association was found for maternal smoking and GP visits for the child's respiratory symptoms: AOR 0.71 (0.51–0.99). The associations between maternal smoking and GP visits for their children, adjusted for different sources of confounding and stratified for the severity of the child's respiratory symptoms are specified in table 4. Especially children with mild respiratory symptoms are less likely to be taken to the GP when their mothers smoke: AOR 0.58 (0.33–1.01).

**Table 3:** Adjusted odds ratio (AOR) and 95% confidence intervals (CI) for the associations between respiratory symptoms or health care use and having a parent who smokes.

	at least one parent smokes	the mother smokes	the father smokes
	AOR (95% CI) *	AOR (95% CI) †	AOR (95% CI) †
**any respiratory symptoms**	1.17 (0.99–1.39)	**1.46 (1.16–1.84)**	0.90 (0.73–1.11)
**mild respiratory symptoms**	**1.27 (1,06–1.52)**	**1.50 (1.19–1.91)**	0.97 (0.78–1.21)
**severe respiratory symptoms**	0.90 (0.71–1.14)	1.03 (0.75–1.40)	0.86 (0.64–1.15)
**GP any reason**	0.95 (0.79–1.14)	0.91 (0.71–1.16)	0.97 (0.78–1.22)
**GP respiratory**	0.92 (0.72–1.18)	**0.71 (0.51–0.99)**	1.20 (0.90–1.60)
**GP non-respiratory**	0.98 (0.79–1.23)	1.15 (0.86–1.55)	0.82 (0.62–1.08)
**Specialized care**	0.91 (0.67–1.22)	1.09 (0.75–1.59)	0.87 (0.61–1.25)

* doctor diagnosed asthma ever, gender of the child, weight at birth, siblings, being breast-fed, and age, ethnicity, education and allergic constitution of the parents. health care use also adjusted for respiratory symptoms

† additional adjustment for smoking by the other parent

**Table 4:** Crude odds ratio (cOR), adjusted odds ratio (AOR) and 95% confidence intervals (CI) for the associations between GP visits in the past two months and having a mother who smokes

	cOR	AOR model 1	AOR model 2	AOR model 3
**GP any reason**	1.06 (0.86–1.31)	1.07 (0.85–1.35)	0.98 (0.77–1.24)	0.91 (0.71–1.16)
**GP respiratory**	0.96 (0.73–1.27)	0.89 (0.65–1.22)	0.79 (0.58–1.10)	**0.71 (0.51–0.99)**
*mild respiratory symptoms*	0.71 (0.44–1.14)	0.64 (0.37–1.08)	0.58 (0.33–1.01)	-
*severe respiratory symptoms*	1.10 (0.69–1.78)	0.90 (0.53–1.54)	0.86 (0.49–1.52)	-
**GP non-respiratory**	1.15 (0.88–1.48)	1.24 (0.93–1.66)	1.19 (0.89–1.60)	1.15 (0.86–1.55)

model 1: adjusted for smoking by the father

model 2: additional adjustment for gender of the child, doctor diagnosed asthma of the child ever, weight at birth, siblings, being breastfed, and age, ethnicity, education and allergic constitution of the parents

model 3: additional adjustment for severity of respiratory symptoms of the child

## Discussion

We found an increased prevalence of respiratory symptoms among children exposed to parental smoking, which is consistent with previous reports [1-7] As expected, maternal smoking had a larger impact on the child's respiratory symptoms and health care use as compared to paternal smoking. Especially mild respiratory symptoms were much more common among children if the mother smoked. Furthermore, children were less likely to be taken to the general practitioner for respiratory symptoms if their mothers smoked. These findings are consistent with the results of a study conducted among children with asthma [18] in which (the amount of) parental smoking was negatively associated with asthma visits and in which maternal smoking (as compared to smoking by the father only) was negatively associated with the number of asthma contacts for the children. This consistency strengthens the validity and generalisability of the hypothesis that mothers who smoke may face barriers in visiting the GP for the child's respiratory symptoms. Mothers who smoke may feel reluctant to visit the GP for the child's respiratory symptoms because they feel guilty or might be afraid to be told to stop smoking. [15,17,18,20]. Our results are only partly in agreement with the assumption that barriers to accessing GP care apply only to routine, preventive care [15], leading to exacerbations requiring acute care)[21]. In our study, the potential underutilization of health care for children with mild respiratory symptoms might indeed suggest postponement of seeking care. However, there was no evidence of associated exacerbations in terms of severe respiratory symptoms or the use of specialized care for respiratory disease among children with mothers who smoked.

Our study has several strengths. The PIAMA study provides longitudinal data on smoking behavior of both parents, information on frequency of several respiratory symptoms of the children, health care utilization of the children with the reason for the visit and information on potential confounders such as social factors [17,22], parental education [23-25]. number of older siblings [17,18,24] and parental allergy [24,25] It is important to be able to account for smoking behavior of the other parent because maternal and paternal smoking have different effects, while smoking habits of both parents are closely related.

The longitudinal aspect of our study is of great importance. In a recent cross-sectional study, hospitalization of children with asthma was inversely associated with parental smoking [8]. One of the explanations given by the authors was that parents may have changed their smoking behavior as a result of the child's hospitalization. A similar reasoning was followed in another cross-sectional study [24]; subjects with aggravated symptoms might avoid exposure to tobacco smoke, thus removing a potential association between ETS and severity of symptoms. To reduce the risk of parental smoking being influenced by respiratory symptoms of the child, we could use exposure data of 1 year previous to the outcome data thus avoiding the possible bias of a cross-sectional design.

Clearly there are also some points of concern. Categories of respiratory symptom severity in our study were based on reported symptoms during the past year, while health care use generally applied to the past two months only. Despite this inconsistency, the results showed that the severity of the child's respiratory symptoms was a strong determinant of health care utilization. Another limitation is that we do not know whether the mother or the father actually visited the GP with the child, or who decided that the GP should be consulted.

Finally, we used only self-reported data for parental smoking, the frequency and reason for health care visits and the severity of the child's respiratory symptoms. Parents may under-report smoking if their children have respiratory symptoms [8]. Had this been the case, this would imply that the associations that we found would in fact even be stronger. However, self-reported smoking has been shown to be reasonably valid within this population [26]. Because of the self reported health care information, the negative association between maternal smoking and health care visits could also be explained if mothers who smoke do not want to admit (report) that they bring children in for respiratory problems. However, we have several reasons to believe that this is not the main conclusion of our findings. First, we did find an increased frequency of self-reported mild respiratory symptoms among children with mothers who smoke, and it seems unlikely that mothers who smoke do admit respiratory symptoms but withhold information on health care use. Secondly, the negative association between maternal smoking and health care visits was stronger for children with mild respiratory symptoms as compared to children with severe symptoms, which makes more sense if the association is real and not the result of under reporting. Finally, we used a comprehensive postal questionnaire (and not a face to face interview) in which smoking and health care use were just two of the many items. For these reasons parents may not be triggered to give 'social desirable' answers.

However, to evaluate whether parents who do or do not smoke equally recognize and value their child's respiratory symptoms and are equally 'truthful' in reporting health care utilization, self-reported measures and objective measures should be compared in further research.

The results of our study have some practical as well as theoretical implications. First, the results stress the importance of protecting children against environmental tobacco smoke exposure. Furthermore, health care workers should be informed about the fact that mothers who smoke may not take their child to the general practitioner in case of (mild) respiratory symptoms. This aspect could be addressed in their education. In the Netherlands all parents regularly attend healthy baby clinics until the child is four years old. These 'preventive' visits provide an excellent opportunity to explicitly inquire after the respiratory health of the child in order to track potential underutilization of care. If necessary, the child can be referred to the GP. Finally, our results also indicate that, as was also discussed by Crombie et al [18], health care utilization cannot be used as a valid proxy measure for respiratory symptom- or disease severity, without taking into account non-clinical factors such as parental smoking.

## Conclusion

Mild respiratory symptoms are much more common among children if the mother smokes. Furthermore, mothers who smoke appear to underutilize health care for their children with mild respiratory symptoms. Health care workers should be informed about this phenomenon. Inquiring after the respiratory health of the children during regular visits to healthy baby clinics may help to track potential underutilization of care.

## Competing interests

The author(s) declare that they have no competing interests.

## Authors' contributions

MJ analyzed and interpreted the data and drafted the manuscript. AW contributed to the coordination of data acquisition, carried out initial data management, and helped in the conception, design and revision of the current study and manuscript. BB, JJ and HS are responsible for the conception, design and coordination of the PIAMA project as a whole and were involved in critically revising the manuscript. CB helped in the conception and design of the study, interpretation of the results and revision of the manuscript. MK made substantial contributions to data acquisition, data management and manuscript revision. All authors read and approved the final manuscript.

## Pre-publication history

The pre-publication history for this paper can be accessed here:


